# Noninvasive factors predicting the maintenance of pregnancy for more than 4 weeks after rescue cerclage in singleton pregnancies with cervical dilatation and prolapsed membrane

**DOI:** 10.1097/MD.0000000000037690

**Published:** 2024-03-29

**Authors:** Ji Kwon Park, Juseok Yang, Hyen Chul Jo, Jong Chul Baek, Ji Eun Park

**Affiliations:** a Department of Obstetrics and Gynecology, Hanyang University Hanmaeum Changwon Hospital, Changwon, Korea; b Department of Obstetrics and Gynecology, Gyeongsang National University Changwon Hospital, Changwon, Korea; c Department of Obstetrics and Gynecology, Gyeongsang National University School of Medicine, Jinju, Korea; d Institute of Health Science, Gyeongsang National University, Jinju, Korea.

**Keywords:** cervical insufficiency, noninvasive markers, preterm birth, rescue cerclage

## Abstract

Studies on noninvasive factors and predicting the maintenance of pregnancy, and those comparing the usefulness of these factors with invasive amniotic fluid markers in predicting the maintenance of pregnancy following rescue cerclage, are lacking. Therefore, this study aimed to determine whether C-reactive protein (CRP) levels, White blood cell (WBC) count, absolute neutrophil count (ANC), and platelet-to-lymphocyte ratio (PLR) in maternal blood, which are noninvasive and readily available clinical markers, can predict the maintenance of pregnancy following rescue cerclage in patients with cervical insufficiency (CI). A total of 142 singleton pregnant women (15–28 wk) who underwent rescue cerclage for CI were retrospectively evaluated. The interleukin (IL)-6 concentration in the amniotic fluid; CRP levels, WBC count, ANC, and PLR in the maternal peripheral blood; and degree of cervical dilatation were evaluated before cerclage. The primary outcome was whether the pregnancy was maintained for >4 weeks after rescue cerclage. Among the 142 patients, prolonged pregnancy for >4 weeks following emergent cerclage was observed in 107 (75.35%), while 35 (24.65%) gave birth within 4 weeks. This study demonstrated that the degree of cervical dilatation at diagnosis; WBC count, ANC, and CRP levels in the maternal peripheral blood; and IL-6 concentration in the amniotic fluid significantly differed between the successful and failure groups (all *P* < .05). The area under the curve (AUC) of the amniotic fluid IL-6 concentration was .795 for the prediction of spontaneous preterm birth within 4 weeks after rescue cerclage. Additionally, the AUC of the CRP level, cervical dilatation, WBC count, ANC, and PLR were .795, .703, .695, .682, and .625, respectively. These findings suggest that the preoperative CRP levels can be considered a useful noninvasive marker comparable to amniotic fluid IL-6 concentration for identifying pregnant women with CI at high risk of spontaneous preterm birth following rescue cerclage.

## 1. Introduction

Complications from preterm birth are the leading cause of death in children aged < 5 years worldwide, with an estimated 900,000 mortalities in 2019.^[1]^ Many survivors face lifelong disabilities, ranging from mild behavioral issues or learning difficulties to severe cerebral palsy, including visual or hearing impairments.

Cervical insufficiency (CI), which is attributed to the incomplete support of pressure by the cervix in the absence of labor pain, can cause the amniotic membrane to seep through the cervix and even reach the vagina. CI accounts for 5% to 15% of pregnancy losses in the second trimester or extreme cases of spontaneous preterm birth (<28 wk).^[2]^ Therefore, rescue cervical cerclage, which is the procedure of putting the amniotic membrane back in place and tightening the expanded cervix, is performed to reverse this situation and maintain pregnancy; however, this procedure can result in premature labor, premature rupture of membranes, or infection within a short period after rescue cerclage. Moreover, more than half of CI cases are associated with subclinical microbial invasion of the amniotic cavity or intra-amniotic inflammation^[3,4]^; therefore, rescue cervical cerclage is not recommended in these cases due to poor prognosis.

Predicting the extent to which cerclage will contribute to pregnancy maintenance is crucial when planning rescue cerclage. To date, studies have mainly focused on the usefulness of positive factors as biomarkers for predicting postoperative prognosis.^[4]^ Therefore, factors such as interleukin (IL)-6 levels in the amniotic fluid are considered a reliable indicator for identifying the presence of inflammation in the amniotic fluid and predicting the outcome of rescue cerclage.^[5]^ However, amniotic fluid analysis is accompanied by amniocentesis, which is an invasive procedure.

Studies on noninvasive factors, including maternal blood indices and predicting pregnancy maintenance and those comparing the usefulness of these noninvasive factors with invasive amniotic fluid markers in predicting pregnancy maintenance following rescue cerclage, are lacking. Therefore, this study aimed to determine the usefulness of a noninvasive factor as a factor predicting the maintenance of pregnancy for >4 weeks following rescue cerclage in singleton pregnancies when compared with the IL-6 concentration in the amniotic fluid.

Furthermore, our study evaluated the utility of noninvasive and readily available factors that can predict the maintenance of pregnancy after rescue cerclage in patients with CI after comparing with the IL-6 concentration in the amniotic fluid.

## 2. Methods

### 2.1. Participants

This retrospective cohort study included 142 singleton pregnant women with cervical dilation and visible prolapsed amniotic membranes who underwent emergency cerclage at the Gyeongsang National University Changwon Hospital, a tertiary referral teaching hospital, from March 2016 to May 2023. The inclusion criteria for emergency cerclage at our facility included visible or prolapsed membranes upon speculum examination. IL-6 concentration in the amniotic fluid was measured by amniocentesis immediately before emergent cerclage. The cohort comprised pregnant women between 15 + 6 and 28 + 0 weeks of gestation with painless cervical dilation of 1 to 6 cm and available outcome data. Furthermore, the exclusion criteria included women who had fetuses with major malformations, active preterm labor, preterm premature rupture of the membranes (PPROM), active vaginal bleeding, clinical chorioamnionitis, and a revision of history- or ultrasound-indicated cerclage.

All patients were treated under general anesthesia using a modified Shirodkar-type purse string cerclage using a 5 mm Mersilene tape (Ethicon, Inc., Somerville, NJ, USA). A Foley catheter balloon was intracervically inserted and gently compressed in certain circumstances when the membranes had prolapsed out of the external cervical os to force the membranes back into the uterus and facilitate suturing. The patients received prophylactic intravenous antibiotics (1 g of cefoxitin 2 times daily or 2 g of ceftriaxone daily) during rescue cerclage, which was continued for at least 5 days. In cases where ureaplasma or mycoplasma colonization from the cervix yielded positive results, 250 mg of clarithromycin was added 2 times daily for 5 days. preoperatively, prophylactic tocolytic agents (ritodrine or atosiban) were administered and maintained for at least 48 hours. After rescue cerclage, intravaginal micronized natural progesterone (Utrogestan® 200 mg) was applied daily and discontinued in cases of ruptured membranes. Prenatal corticosteroids were administered for fetal lung maturation in cases of labor onset or ruptured membranes, which were considered an indication for preterm removal of the cerclages; otherwise, the cerclages were electively removed at 36 gestational weeks. In cases of PPROM without labor during pregnancy, antibiotics (1 g of cefoxitin 2 times daily or 2 g of ceftriaxone and 250 mg of clarithromycin 2 times daily) were used for >10 days.

All participants’ medical records were examined, and information on the demographics and obstetric results was obtained from the hospital database. Maternal peripheral blood collection and vaginal swabs were performed at admission before the administration of the antibiotics, corticosteroids, or tocolytics. White blood cell (WBC), neutrophil, lymphocyte, and platelet counts were detected using flow cytometry. The neutrophil-to-lymphocyte ratio was defined as the absolute neutrophil count (ANC) divided by the absolute lymphocyte count. C-reactive protein (CRP) levels were measured using an immunoturbidimetric assay. The detection of cervical ureaplasma and mycoplasma colonization in the cervix was performed using a commercially available Sexually Transmitted Infections-12 Kit (STD-12 GC Labs, Seoul, Republic of Korea) based on multiplex real-time polymerase chain reaction. Amniotic fluid samples were obtained via ultrasound-guided transabdominal amniocentesis for the measurements of glucose concentration, IL-6 concentration, and culture for aerobic, anaerobic, and genital mycoplasmas.

Each variable was compared by categorizing the patients into 2 groups based on whether they maintained gestation for 4 weeks after rescue cerclage. The continuous variables from patient information were compared using analysis of variance and the Kruskall–Wallis test. Additionally, the area under the receiver operating characteristic (ROC) curve (AUC) was used to evaluate the predictive efficiency of each marker for predicting the maintenance of pregnancy ≥4 weeks after rescue cerclage. The results were presented as odds ratio and 95% confidence interval, and significant differences were considered at *P* < .05 for the statistical tests. All statistical analyses were conducted using Web-R ver. 3.4.1 (http://web-r.org), a web-based statistical analysis program. This study protocol was approved by the Ethics Committee of Gyeongsang National University Hospital, and the requirement for consent to participate was waived due to its retrospective design (IRB Number: GNUCH 2024-01-13). All study procedures performed involving human participants followed the ethical standards of the institutional and national research committee and the 1964 Helsinki Declaration and its later amendments or comparable ethical standards.

## 3. Results

A total of 142 patients were included in this study. Table 1 presents an overview of the clinical and maternal peripheral blood laboratory characteristics of the study population. The mean gestational age at the time of rescue cerclage and delivery was 22.2 ± 3.2 and 33.4 ± 4.0 weeks, respectively. Additionally, the mean prolongation of pregnancy after emergent cerclage was 11.5 ± 5.6 weeks.

**Table 1:** Clinical characteristics of the study population according to the outcome of predicting the maintenance of pregnancy after rescue cerclage.

	Maintaining pregnancy <4 wk after rescue cerclage	Maintaining pregnancy ≥4 wk after rescue cerclage	Total	*P*-values
	(N = 35)	(N = 107)	(N = 142)	
Maternal age (yr)	33.6 ± 5.0	33.2 ± 3.8	33.4 ± 4.0	.643
Height (cm)	161.8 ± 5.6	161.0 ± 4.5	161.4 ± 4.9	.467
Prepregnancy BMI (kg/cm^2^)	24.5 ± 3.9	22.9 ± 4.2	23.6 ± 4.7	.142
BMI at cerclage placement (kg/cm^2^)	26.6 ± 3.8	24.7 ± 4.1	25.3 ± 4.5	.061
Cervical ureaplasma or mycoplasma (%)	15 (42.9%)	31 (29.0%)	46 (32.4%)	.124
**Cervical dilatation at diagnosis (cm**)	**3.2 ± 1.6**	**2.2 ± 0.9**	**2.4 ± 1.2**	**.015**
**WBC (× 10^3^/µL**)	**12.3 ± 3.4**	**10.1 ± 2.4**	**10.5 ± 2.8**	**.014**
Segmented neutrophil (%)	73.4 ± 7.3	74.0 ± 7.3	73.8 ± 6.9	.721
**ANC (× 10^3^/µL**)	**9.2 ± 2.9**	**7.6 ± 2.1**	**7.8 ± 2.5**	**.006**
Lymphocyte (%)	18.3 ± 5.0	18.7 ± 8.2	18.6 ± 6.8	.774
NLR	5.2 ± 1.5	4.5 ± 1.9	4.5 ± 2.1	.731
Platelet (× 10^3^/µL)	261.4 ± 57.1	256.6 ± 48.9	260.9 ± 55.0	.698
**PLR**	**126.9 ± 37.4**	**155.0 ± 49.6**	**149.9 ± 47.1**	**.021**
**CRP (mg/L**)	**10.7 ± 6.5**	**6.5 ± 12.3**	**8.4 ± 18.6**	**.035**
**IL-6 (pg/mL**)	**3566.6 ± 1999.7**	**1317.3 ± 1573.7**	**1647.6 ± 1817.1**	**<.001**
Amniotic fluid glucose (mg/dL)	28.4 ± 21.5	35.5 ± 14.4	34.2 ± 16.2	.119
**Positive amniotic fluid culture (%**)	**9 (25.7%**)	**4 (3.7%**)	**13 (9.2%**)	**<.001**
Gestational age at rescue cerclage (wk)	21.6 ± 2.9	22.6 ± 2.5	22.2 ± 3.2	.148
**Gestational age at delivery (wk**)	**23.3 ± 3.2**	**35.4 ± 3.1**	**33.4 ± 5.9**	**<.001**
**PPROM after rescue cerclage** (%)	**20 (57.1%**)	**19 (26.2%**)	**39 (27.5%**)	**<.001**
**Cerclage to delivery interval (wk**)	**1.8 ± 1.3**	**13.4 ± 4.1**	**11.5 ± 5.6**	**<.001**

Values are presented as the means ± standard deviations for continuous variables and n (%) for categorical variables, ANC = absolute neutrophil count, CRP = C-reactive protein, IL-6 = interleukin-6, NLR = neutrophil-to-lymphocyte ratio, PLR = platelet-to-lymphocyte ratio, PPROM = preterm premature rupture of membrane, WBC = White blood cell.

The bold values are values with significant differences (*p* < 0.05).

Among the 142 women, prolonged pregnancy for >4 weeks after emergent cerclage was observed in 107 (75.35%), and 35 (24.65%) gave birth within 4 weeks. No significant differences were observed in the maternal age, height, body mass index, and gestational age at cerclage between the groups. The degree of cervical dilatation at diagnosis; WBC count, ANC, and CRP levels in the maternal peripheral blood; and IL-6 concentration and positive culture in the amniotic fluid were significantly higher, and the platelet-to-lymphocyte ratio (PLR) value was significantly lower in the group that did not maintain pregnancy for >4 weeks than in the group that maintained pregnancy for >4 weeks. However, no significant differences were found in the neutrophil, lymphocyte, platelet counts, and glucose concentration in the amniotic fluid between the groups. Additionally, the incidence of PPROM after rescue cerclage was significantly higher in the group that did not maintain pregnancy for >4 weeks than in the group that maintained pregnancy for >4 weeks (57.1% vs 26.2%, *P* < .001).

**Figure 1.**
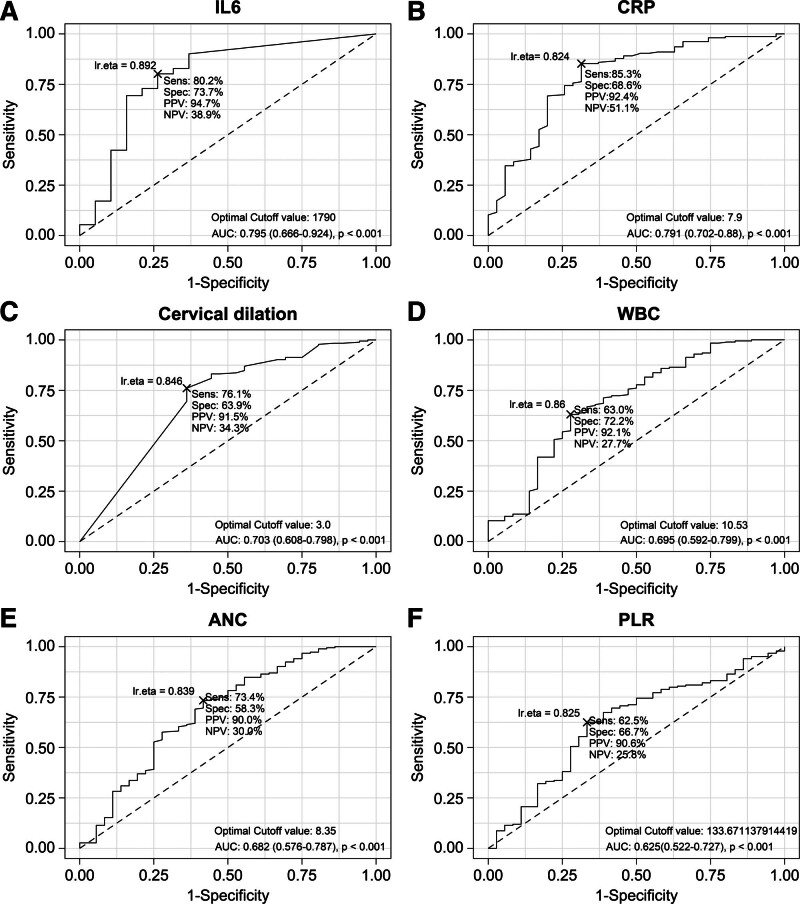
Cutoff, using the optimal marker level, to predict maintaining pregnancy ≥ 4 weeks after rescue cerclage according to the ROC curve. (A) IL-6 level; (B) CRP level; (C) cervical dilatation; (D) WBC; (E) ANC; (F) PLR. ANC = absolute neutrophil count, CRP = C-reactive protein, IL-6 = interleukin-6, PLR = platelet-to-lymphocyte ratio, ROC = receiver operating characteristics, WBC = White blood cell.

Using the ROC curve analysis, we evaluated the predictive power of the serum CRP levels, WBC count, ANC, PLR, and degree of cervical dilatation in maintaining pregnancy for >4 weeks after rescue cerclage compared to the IL-6 concentration in the amniotic fluid (Table 2). Among them, IL-6 concentrations and CRP levels demonstrated an AUC value of .795 and .791, respectively. The degree of cervical dilatation showed the next highest AUC value of .703. Figure 1 illustrates the ROC curves of the 3 markers. The cutoff values for predicting prolonged pregnancy beyond 4 weeks associated with rescue cerclage were 1790, 7.9, and 3.0 cm for IL-6 concentrations, CRP levels, and the degree of cervical dilatation.

**Table 2:** ROC curves and best cutoff points with its sensitivity, specificity, PPV, and NPV for each marker predicting maintaining pregnancy ≥4 weeks after rescue cerclage.

	ROC-AUC (95% CI)	Sensitivity	Specificity	PPV	NPV	Optimal cutoff
IL-6	0.795 (0.666–0.924)	80.2	73.7	94.7	38.9	1790
CRP	0.791 (0.702–0.88)	85.3	68.6	92.4	51.1	7.9
WBC	0.695 (0.592–0.799)	63.0	72.2	92.1	27.7	10.53
ANC	0.682 (0.576–0.787)	73.4	58.3	90.0	30.0	8.35
PLR	0.625 (0.522–0.727)	62.5	66.7	90.6	25.8	133.67
Cervical dilatation at diagnosis	0.703 (0.608–0.798)	76.1	63.9	91.5	34.3	3.0

ANC = absolute neutrophil count, CI = confidence interval, CRP = C-reactive protein, IL-6 = interleukin-6, NPV = negative predictive value, PLR = platelet-to-lymphocyte ratio, PPV = positive predictive value, ROC = receiver operating characteristics, WBC = White blood cell.

## 4. Discussion

The main finding in this study was that IL-6 concentration in the amniotic fluid showed the best predictive power in predicting pregnancy for >4 weeks after rescue cerclage, and CRP levels in the maternal blood showed predictive power comparable to that of the IL-6 concentration in the amniotic fluid and higher sensitivity. Additionally, maternal blood WBC count, ANC, and PLR and the degree of cervical dilatation at rescue cerclage showed significant differences according to whether pregnancy was maintained for >4 weeks after rescue cerclage.

Intra-amniotic inflammation, which is observed in approximately 80% of CI cases, has consistently been reported as a strong risk factor for poor pregnancy outcomes.^[4,6]^ Therefore, some clinicians are hesitant to perform rescue cerclage for dilated cervix; moreover, the criteria for deciding whether to perform rescue cerclage remain unclear. Some studies support the use of amniocentesis to determine the presence of inflammation before performing cerclage^[7]^; moreover, high IL-6 concentrations through amniocentesis are reportedly associated with a short pregnancy duration and poor pregnancy prognosis.^[5,8]^ These findings are consistent with those reported in our study. An IL-6 concentration of ≥1790 was found to be a risk factor for not maintaining the pregnancy for >4 weeks after rescue cerclage.

IL-6 in the amniotic fluid is a reliable indicator for predicting CI prognosis; however, it has limited clinical application due to the need for invasive manipulation, which could result in amniotic membrane rupture or bleeding. Therefore, finding new, reliable, and noninvasive indicators for predicting the pregnancy outcomes of rescue cerclage is crucial.

Kim et al^[9]^ reported AUC values of .686, .691, and .693 for maternal plasma insulin-like growth factor binding protein-3 (IGFBP-3), macrophage inflammatory protein-1 alpha (MIP-1α), and S100A8/A9, respectively, for predicting spontaneous preterm birth of <28 weeks after rescue cerclage; therefore, IGFBP-3, MIP-1α, and S100A8/A9 were considered noninvasive independent biomarkers. However, IGFBP-3, MIP-1α, and S100A8/A9 detection tests, which are limited to laboratories, are not easily accessible for clinical use. Moreover, Lin et al^[10]^ reported changes in the inflammation levels (calculated using platelet, neutrophil, lymphocyte, and monocyte counts) in the maternal peripheral blood before and after rescue cerclage to be beneficial in predicting the maintenance of pregnancy. However, since these blood test results are performed after rescue cerclage, their use in deciding whether to perform cerclage is challenging; moreover, the need for further calculations is a limitation.

In this study, a maternal serum CRP level of >7.9 mg/L immediately before cerclage was considered a predictor of failure to maintain pregnancy for >4 weeks after rescue cerclage. A CRP level of >5 mg/L has been demonstrated as a risk factor for predicting childbirth before 28 weeks of pregnancy after rescue cerclage^[11]^; however, this study differed from our study as it included prophylactic and ultrasound-indicated cerclage in addition to rescue cerclage.

The indications for rescue cerclage vary among each society. The American College of Obstetricians and Gynecologists^[12]^ recommends rescue cerclage for cervical dilatation in the second trimester of pregnancy; however, the Royal College of Obstetricians and Gynaecologists^[13]^ and the Society of Obstetricians and Gynaecologists of Canada^[14]^ recommends its use in dilatations of <4 cm. Our study results demonstrated that pregnancy could be maintained for >4 weeks after performing emergent cerclage when dilatation was <3 cm.

The strength of our study is that it targeted a relatively large study group (n = 142) that underwent rescue cerclage at a single institution, and it compared the IL-6 concentrations (through invasive amniocentesis) and noninvasive biomarkers as predictors of maintaining pregnancy for >4 weeks after rescue cerclage.

This study had certain limitations. First, it was retrospective and was conducted at a single institution. Second, no external validation of our findings was performed. Third, the study included participants over 8 years. Lastly, all women with CI undergoing rescue cerclage were not provided uniform treatment, particularly in the type of antibiotics used. Therefore, further well-designed prospective studies on larger patient cohorts are warranted to confirm these findings.

In conclusion, this study demonstrated that maternal peripheral blood CRP levels may be a useful noninvasive marker comparable to IL-6 concentration in the amniotic fluid in predicting the maintenance of pregnancy beyond 4 weeks after rescue cerclage. A high possibility of maintaining pregnancy for >4 weeks after rescue cerclage was observed in cases of cervical dilatation <3 cm. Therefore, these results may be helpful to obstetricians in the initial selection of patients undergoing rescue cerclage for CI and subsequent clinical management and counseling of these patients due to their noninvasive, convenient, and fast obtainable nature of clinical information.

## Author contributions

**Conceptualization:** Ji Kwon Park, Ji Eun Park.

**Data curation:** Juseok Yang, Hyen Chul Jo, Jong Chul Baek.

**Formal analysis:** Ji Kwon Park, Ji Eun Park.

**Writing – original draft:** Ji Kwon Park, Ji Eun Park.

**Writing – review & editing:** Ji Eun Park.
